# PbS and PbO Thin Films via E-Beam Evaporation: Morphology, Structure, and Electrical Properties

**DOI:** 10.3390/ma15196884

**Published:** 2022-10-04

**Authors:** Saad Akhtar, Nimra Saeed, Muhammad Bilal Hanif, Zia-ur-Rehman, Salahuddin Dogar, Waqar Mahmood, Michał Mosiałek, Bogna Daria Napruszewska, Muhammad Ashraf, Martin Motola, Abdul Faheem Khan

**Affiliations:** 1 Department of Materials Science and Engineering, Institute of Space Technology,1-National Highway, Islamabad 44000, Pakistan; 2 Department of Inorganic Chemistry, Faculty of Natural Sciences, Comenius University in Bratislava, Ilkovicova 6, Mlynska Dolina, 842 15 Bratislava, Slovakia; 3 C-CET, H-11, NESCOM, Islamabad 44000, Pakistan; 4 Material Synthesis and Characterizations (MSC) Laboratory, Department of Physics, Fatima Jinnah Women University (FJWU), The Mall, Rawalpindi 46000, Pakistan; 5 Jerzy Haber Institute of Catalysis and Surface Chemistry PAS, Niezapominajek 8, 30-239 Krakow, Poland; 6 Optics Laboratories, P.O. Nilore, Islamabad 45500, Pakistan

**Keywords:** thin films, lead oxide, lead sulfide, electron beam evaporation, Rutherford backscattering, band gap

## Abstract

Thin films of lead sulfide (PbS) are being extensively used for the fabrication of optoelectronic devices for commercial and military applications. In the present work, PbS films were fabricated onto a soda lime glass substrate by using an electron beam (e-beam) evaporation technique at a substrate temperature of 300 °C. Samples were annealed in an open atmosphere at a temperature range of 200–450 °C for 2 h. The deposited films were characterized for structural, optical, and electrical properties. Structural properties of PbS have been studied by X-ray diffraction (XRD), field emission scanning electron microscopy (FESEM), energy dispersive spectroscopy (EDS), and Rutherford backscattering spectrometry (RBS). The results of XRD showed that the PbS thin film was crystalline in nature at room temperature with cubic crystal structure (galena) and preferential (111) and orientation (022). The morphology of the thin films was studied by FESEM, which also showed uniform and continuous deposition without any peel-off and patches. EDS analysis was performed to confirm the presence of lead and sulfur in as-deposited and annealed films. The thickness of the PbS film was found to be 172 nm, which is slightly greater than the intended thickness of 150 nm, determined by RBS. Ultraviolet-Visible-Near-Infrared (UV-Vis-NIR) spectroscopy revealed the maximum transmittance of ~25% for as-deposited films, with an increase of 74% in annealed films. The band gap of PbS was found in the range of 2.12–2.78 eV for as-deposited and annealed films. Hall measurement confirmed the carriers are p-type in nature. Carrier concentration, mobility of the carriers, conductivity, and sheet resistance are directly determined by Hall-effect measurement. The as-deposited sample showed a conductivity of 5.45 × 10^−4^ S/m, which gradually reduced to 1.21 × 10^−5^ S/m due to the composite nature of films (lead sulfide along with lead oxide). Furthermore, the present work also reflects the control of properties by controlling the amount of PbO present in the PbS films which are suitable for various applications (such as IR sensors).

## 1. Introduction

Lead sulfide (PbS) is an inorganic semiconductor and is found naturally in the form of minerals called galena [1]. It has been found that PbS shows strong quantum confinement effects, having a high dielectric constant and admiring electrical conductivity at room temperature (i.e., 10^−3^ S m^−1^), which makes it a promising material for optoelectronic applications [2]. PbS is an important semiconductor in group IV–VI of the periodic table with a narrow band gap of 0.41 eV [3] and has a large excitation Bohr’s radius of 18 nm [4]. The effective quantum confinement is directly related to the thickness of the film. By varying the thickness of the film, the PbS band gap can be tuned and due to p-type conduction, its direct band gap varied by up to 3 eV. In contrast, the indirect narrow band gap found 0.41 eV suitable for detectors at room temperature [5,6]. Hence, it can be utilized in applications such as optoelectronic circuits, optical devices, and solar cells [7,8]. Therefore, potential interest rapidly increased for the preparation of PbS thin films by different techniques. Authors have reported different results based on the implementation techniques by using atomic layer deposition (ALD) [9], solid vapor deposition [10], thermal evaporation method [11], RF sputtering [12], spray pyrolysis [13], and electro-deposition [14].

Traditionally, most thin films have been prepared by the chemical bath deposition (CBD) method [15], which has the limitation of thickness. The minimum thickness of 300–400 nm can be achieved; however, impurities and defects are found on the surface of the film. At the time of synthesis and deposition, by-products are mainly added in chemical reactions; these by-product impurities lead to undesirable effective results in the photosensitivity of the film and cause a problem in photoactive and optoelectronic devices. Thus, physical vapor deposition (PVD) plays a significant role in the thin film’s production and very low ionic dissociation is formed. The electron beam evaporation technique has been used for depositing the thin film composite structure on soda lime glass substrate. The main reason for choosing the e-beam technique instead of other techniques such as magnetron sputtering, pulsed laser deposition technique, atomic layer deposition, molecular beam epitaxy, etc. is because they are quite expensive techniques. Moreover, the composite structure of PbS and PbO can be easily obtained in the e-beam technique, which can be challenging with other techniques. E-beam is a relatively inexpensive technique to produce composite structure films, which is the requirement of the industry for concerned applications. In contrast, we have deposited PbS thin films by the electron beam (e-beam) evaporation method to acquire a high deposition rate with good uniformity of films and without any significant impurities. Hence, physical deposition methods are relatively superior to chemical methods. The films deposited by the e-beam technique show better adhesion properties and are uniformly conformal and pinhole-free. The feature of the growth rate of thin film and its size can be the yield, by adjusting the reaction time and temperature, though it is a challenging task to obtain the desired optical properties. Therefore, e-beam continued the exploration of thin film crystal quality improvement, which is not only exciting but also a diverse ongoing research field [16,17,18,19].

In this research work, PbS thin films have been deposited by the e-beam technique with ~172 nm thickness and annealed in an open atmosphere for 2 h. The films’ structural, optical, and electrical properties have been characterized using various equipment. The deposited films show a composite structure containing PbS and PbO, which may be employed for the application of IR sensors [18].

## 2. Materials and Methods

The target material of PbS (Sigma Aldrich, MO, USA; CAS No. 1314-87-0) with a purity level of ~99.9% was used for deposition purposes. The films were deposited onto the soda lime glass substrate (CAS No. 65997-17-3, Merck, MO, USA). Before deposition, substrates were washed ultrasonically, cleaned, and dried with acetone followed by amyl acetate to remove native oxide layers. Neat substrates were loaded onto the sample holder inside the chamber for deposition. The pallets were made by using PbS powder and placed in a molybdenum crucible inside the chamber for deposition [12]. 

The deposition of PbS and PbO was performed using the Edwards coating system for improved uniformity and homogeneity of films. Before deposition, the vacuum was set to less than 1 × 10^−5^ mbar. The substrate temperature was maintained at 300 °C and the rotation speed was set at 30 rpm. The substrate was positioned 35 cm away from the source. The base pressure was maintained at ~1 × 10^−5^ mbar and the deposition was performed at a rate of ~0.15 nm/s. The thickness of the films was monitored using a crystal quartz detector during deposition. The total thickness of 150 nm was deposited in this way. The electron beam evaporation technique involves surface melting of the target material before deposition. During melting, the sulfur dissociates from Pb and moves towards substrates. Some of the PbS atoms reach and condense at the substrate, while some Pb atoms react with oxygen (residual oxygen in the chamber and also due to degassing at low vacuum) present in the chamber. That is why it is relatively easy to fabricate the composite PbS and PbO films using the e-beam technique. Afterward, the samples were annealed at various temperatures of 200 °C, 300 °C, 400 °C, and 450 °C for 2 h to obtain uniformity, removing surface roughness and fine stoichiometry. The structure of these thin films was determined by X-ray diffraction (XRD) using a PANalytical X’Pert Pro system (Malvern B.V., Lelyweg 1, The Netherlands) with the monochromatic Cu Kα at 40 kV and 30 mA in a 2-theta range of 20 to 80°. The surface morphology of the films was investigated by FESEM (MIRA3 TESCAN). The thickness and composition of the films were measured by Rutherford backscattering spectrometry (RBS). The measurements were performed using a 5UDH-2 Pelletron (5 MV Pelletron Tandem Accelerator) with He^2+^ beam. The average energy of the beam was 2 MeV. The analysis was performed by keeping the scattering angle at 170°. The samples were placed at 70° with respect to the incident beam. All RBS measurements were made using Cornell geometry.

Optical transmittance of the as-deposited and annealed films was recorded at room temperature by a Perkin Elmer UV/VS/NIR Lambda 19 Spectrophotometer (spectral resolution for UV-Vis ≤ 0.05 nm and NIR ≤ 0.2 nm) in the wavelength range 200–2500 nm. Electrical conductivity, charge mobility, and sheet resistance of the films were directly measured at room temperature using Hall measurements.

## 3. Results and Discussion

**Figure 1:**
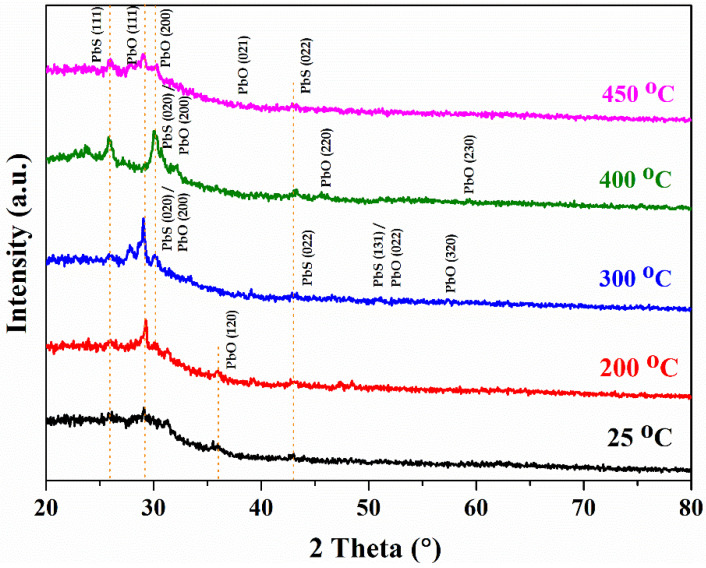
X-ray diffraction pattern of PbS thin film at 25 °C, 200 °C, 300 °C, 400 °C, and 450 °C.

A simple tape test was performed on the films. The PbS thin films were deposited onto the soda lime glass substrate, which was found to be physically stable, with no-peel off or blisters present on the films. Figure 1 shows the PbS thin film of as-deposited and annealed samples. The as-deposited PbS film is crystalline in nature, having a cubic structure along with space group Fm-3m, with a JCPDS card No. 96-231-1041 and 96-900-8695 for PbO and PbS, respectively [20]. The as-deposited films show a minor amount of PbO formed during deposition. The deposition was performed at a relatively high vacuum (10^−5^ mbar); however, due to residual gas and degassing from the chamber, PbO was formed in the films. Furthermore, annealing at elevated temperatures in the open atmosphere also favors the formation of PbO, which is clear from the XRD patterns. It is noticeable that the plane (111) and (200) of PbO are present in all samples. It is also observed that PbO increases with an increase in annealing temperature, which is clear from the XRD patterns. However, the appearance of the plane (111) and (022) also confirmed the presence of PbS. Moreover, with annealing, the film’s crystallinity also improves, as depicted by the XRD result. Above 400 °C, a sufficient quantity of PbO is formed in the films due to the dissociation of sulfur from PbS (due to the melting of PbS in the crucible) and the formation of PbO due to the surplus amount of oxygen from the environment. 

**Figure 2:**
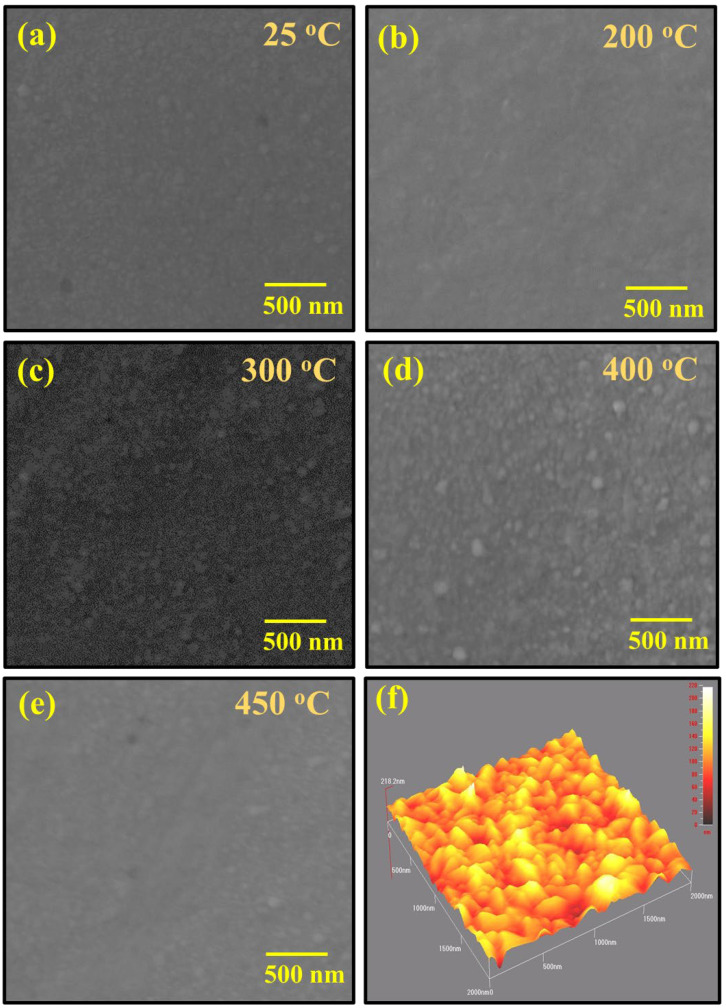
FESEM images of PbS thin film at (**a**) 25 °C, (**b**) 200 °C, (**c**) 300 °C, (**d**) 400 °C, and (**e**) 450 °C, along with the atomic force microscopy topography of as-deposited samples (**f**).

Figure 2 shows the FESEM images of as-deposited and annealed PbS thin film demonstrating a uniform and dense deposition of PbS over the entire substrate. The uniformity further increases as the annealing temperature increases to 300 °C (Figure 2b,c); however, a small radical change in the composition of the films has been observed at 300 °C and 400 °C due to additional oxygen that causes the content of PbO to enhance more rapidly (Figure 2c,d). The film is still intact with the substrate and some round spheres/nanoparticles of PbO have been observed on the surface in the matrix of PbS. The particle size lies in the range of 20–30 nm, as shown in Figure 2c. As the annealing of the sample was performed in an open atmosphere, the lead (Pb) is partly oxidized and become lead oxide (PbO). Above 400 °C, the enhanced formation of PbO has been observed, which also increases its transparency (Figure 2d). In short, all the samples (as-deposited and annealed) show a smooth surface with much less roughness (although the roughness was not measured directly from AFM), with dense and compact films [21]. Furthermore, electron beam evaporation is a feasible technique for the deposition of PbS films, while composite PbS and PbO films can also be obtained by this method [22]. 

Atomic force microscopy (AFM) was used to gather further information related to the surface roughness of as-deposited thin film. The results of AFM show that the surface roughness of the as-deposited sample is approximately ~218 nm and that the samples are quite homogeneous. Generally, the surface roughness reduces with increasing annealing temperature and increases homogeneity (AFM on annealed samples was not performed).

**Figure 3:**
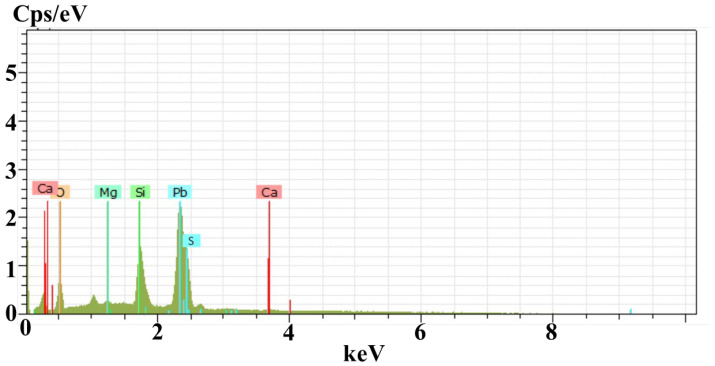
EDS spectra of PbS thin film of as-deposited sample.

Figure 3 shows the results obtained from EDS analysis, with the appearance of major peaks of lead (Pb) and sulfur (S) confirming the presence of PbS thin film. Additionally, the peaks of Si, O, Ca, and Mg elements appear from the substrate. From the tabular data of EDS, the weight percentage of lead is evaluated as 75.13%, whereas sulfur is found to be 5.45% and oxygen is 6.45% at room temperature. As the temperature increases to 300 °C, the amount of oxygen increases with the decrease of sulfur content. The additional oxygen diffusing from the environment leads to the formation of PbO, which is consistent with the XRD results. The contents of other elements comparatively change due to the phenomena of annealing and are provided in Table 1.

**Figure 4:**
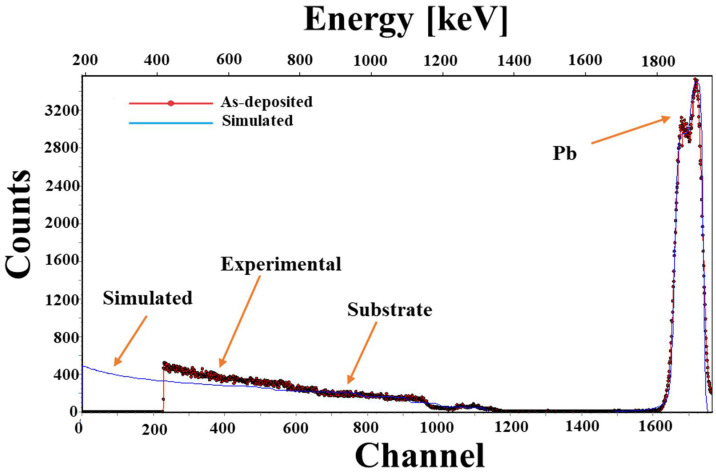
RBS spectra of PbS thin film of as-deposited sample.

Figure 4 shows the experimental versus simulated RBS spectra of PbS thin film at room temperature, which is deposited onto the glass substrate. A single strong peak is evident in the spectrum and is related to Pb, which corresponds to backscattering from Pb atoms. Effective thickness is related to elastic recoil, which is proportional to the thickness of the films [23].

The spectrum shows that the sample was divided into two layers comprising PbS and PbO. The concentration of elements was found to be 95% lead and 5% sulfur in the first layer (adjacent to the substrate). The thickness of the first layer (PbS) was found to be 112 nm. The second layer (PbO), as shown in Figure 4 (shoulder peak), indicates that the concentration is 58% lead and 37% oxygen, with sulfur’s concentration remaining the same (i.e., 5%) in the as-deposited sample. The thickness of the second layer was found to be 60 nm. The overall thickness of the layers was determined to be 172 nm, which is slightly larger than the intended thickness of 150 nm. An insignificant rise in thickness is due to limitations of the crystal quartz monitor during deposition. The third layer consists of the glass substrate. Hence, it can be concluded from the above findings that the deposited film contains PbS as well as PbO due to the addition of a sufficient amount of oxygen during deposition and annealing at elevated temperatures.

In general, for the application of IR detectors, it is necessary to control the absorption of light within a specific range; for this purpose, the optical band gap adjusts in such a manner to achieve the required properties. It is usually a tedious process when tailoring the semiconducting material [24] properties for some specific applications, for which band gap engineering is an essential element.

**Figure 5:**
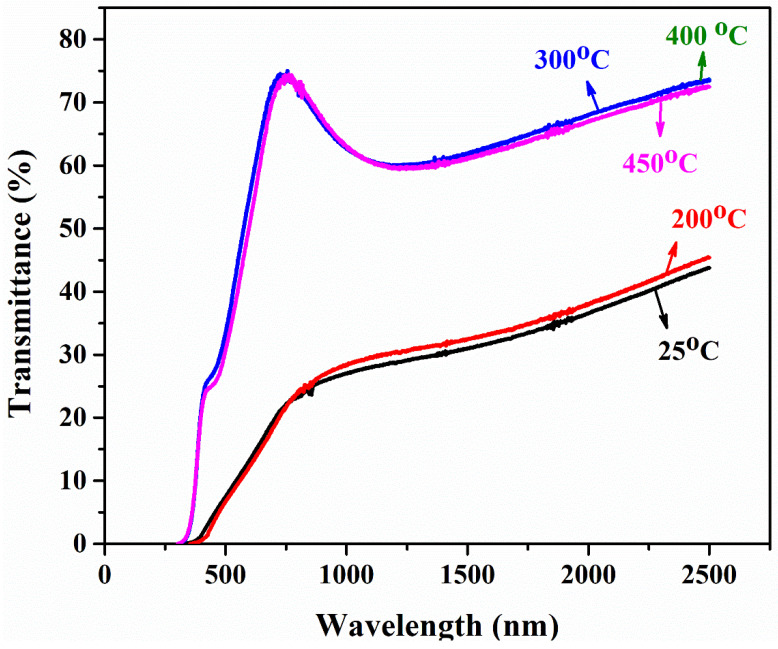
Transmittance spectra of PbS thin film at 25 °C, 200 °C, 300 °C, 400 °C, and 450 °C.

Figure 5 shows the transmittance spectra of as-deposited and annealed PbS thin film in the wavelength range up to 2500 nm. The transmittance of the as-deposited and annealed sample at 200 °C was found to be 25%, with a gradual rise in transmittance observed at a higher wavelength. Kotadiya et al. [25] took a similar measurement of transmittance on PbS films and showed transmittance in the range of ~24% in the visible region, while Seghaier et al. [5] demonstrated transmittance below 50% in the range of 250–850 nm wavelength. As the temperature increased up to 300 °C, a drastic change of increase in the transmittance curve was observed in the visible region, which may be due to the addition of oxygen through annealing the samples in an open atmosphere. It is well known that metal oxides are transparent to visible light, which is also evident in the present case due to PbO’s change of color from black to light yellow. A moderate change after 300 °C has also been observed in the visible and near IR wavelength; this might be ascribed to the profoundly crystalline nature, uniform deposition of thin film, and change of morphology [12].

For all films near the IR and IR region, the transmittance is high, as shown in Figure 5, with a decrease in the transmittance graph noticed, which is due to the interference effect [26,27]. Moreover, the increase in transmittance in the IR region is primarily attributed to the formation of PbO. 

The temperature has a strong influence on the optical properties of semiconducting materials. The same phenomena for PbS are noted by Pretha et al. [28] with heat treatment. In optical properties, band gap plays a vital role in the thin film study, leading to device formation. When a photon hits the electrons, it is absorbed by the valence electrons and the electrons move to the conduction band due to this energy. These excited electrons can be used for any optoelectronic devices (IR detectors), once the electron reaches the minimum energy level of the excited state. For this energy estimation, the band gap is a primary factor to be accurately determined [29]. In this regard, Tauc’s plot is helpful for determining the band gap of semiconducting material:

(1) $${\left(\alpha hv\right)}^{2}=B{\left(hv-E_{g}\right)}^{2}\gamma$$

In the above equation, *E_g_* is the optical band gap, h is Planck’s constant, *v* is the frequency of incident photons, B is a constant called the band tailing parameter, and γ is the index, which can have different values (2, 3, 1/2, and 1/3) corresponding to indirect allowed, indirect forbidden, direct allowed, and direct forbidden transitions, respectively [30]. 

**Figure 6:**
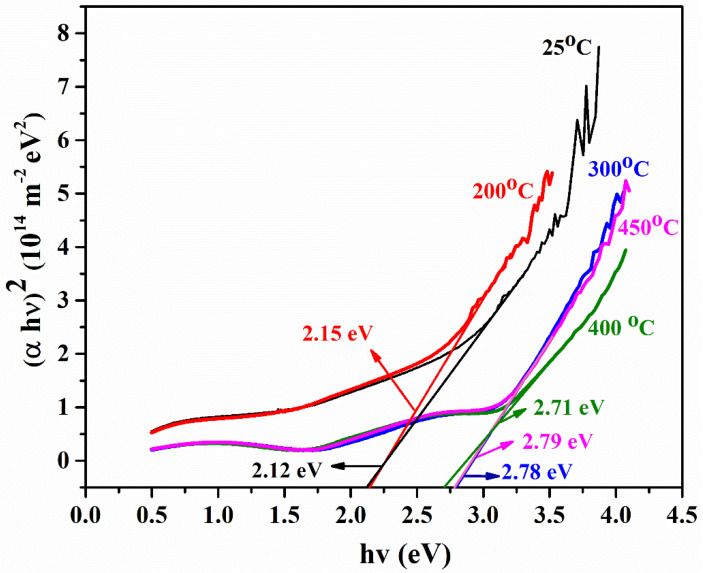
Band gap of PbS thin film at 25 °C, 200 °C, 300 °C, 400 °C, and 450 °C.

The band gap of as-deposited and annealed PbS films are shown in Figure 6. The band gap is observed to increase with increasing temperature. The band gap of bulk PbS is 0.4 eV [31]. However, the band gap in the current study is sufficiently larger (2.12 eV) than the bulk value. This difference in band gaps is due to the following reasons: firstly, this large difference might be due to oxide formation along with PbS [32]; secondly, the lower thickness of the current films compared to the bulk material; thirdly, the effect of quantum confinement in PbS films. In any case, the synergistic effect is the increase in the band gap in PbS films.

The rise in band gap with annealing temperature is attributed to structural modifications, reduction in defects density, increase in oxygen content, and quantum confinement in annealed PbS films [33,34].

The electrical properties of the semiconductors are extremely important with reference to device formation, and these properties are strongly dependent on temperature, thickness, impurity, defects density, etc. [35].

The electrical properties of PbS thin films are calculated using the Van der Pauw approach [36] by keeping a constant current of 1 × 10^−9^ A and a magnetic field of 0.556 T. Ecopia Hall Measurement System 5000 is used to measure the electrical characteristics of PbS thin films at 25 °C. Conductivity, mobility, and sheet resistance are directly measured by using the Hall effect method. 

**Figure 7:**
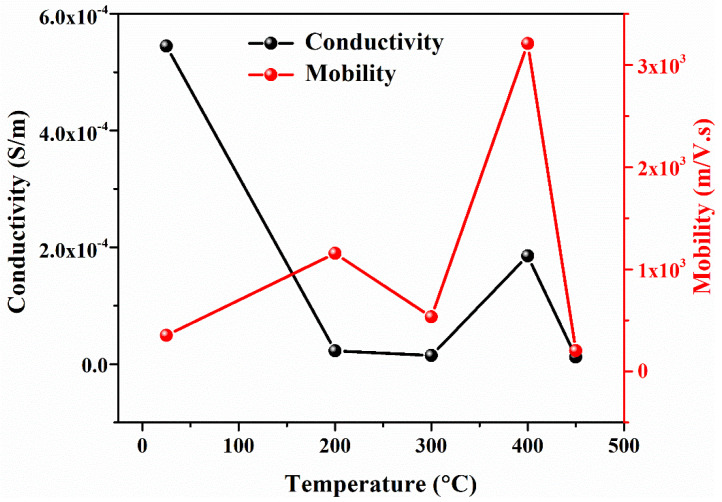
Conductivity and Mobility of PbS at 25 °C, 200 °C, 300 °C, 400 °C, and 450 °C.

The conductivity of as-deposited and annealed PbS films at 200 °C, 300 °C, 400 °C, and 450 °C were found to be 5.45 × 10^−4^, 2.25 × 10^−5^, 1.47 × 10^−5^, 1.85 × 10^−4^, and 1.21 × 10^−5^ S/m, respectively. The conductivity of the as-deposited sample is relatively high due to more charge carriers. With the increase in temperature from 200 °C to 300 °C, the conductivity reduced due to oxide formation (as is already clear from the XRD, SEM, and EDS results), causing a hindrance to the flow of electrons, as shown in Figure 7. However, a slight increase in conductivity was observed at 400 °C, which may be due to an increase in charge carriers’ concentration and their mobility. PbO formation at this temperature is sufficiently high compared to lower temperatures but, at the same time, mobility and charge carriers’ density increase at this temperature, ultimately causing the conductivity to rise. Above 400 °C, the mobility and density of charge carriers reduces with an increase in PbO, causing a decrease in conductivity.

Sheet resistance, also known as surface resistivity, is the resistance measured across the surface of two-dimensional thin film, when the electrodes made contact with the surface of the film [37]. The distance between contacts has no effect on sheet resistance; therefore, it is extensively used to compare the electrical properties of the devices made by different films [38].

**Figure 8:**
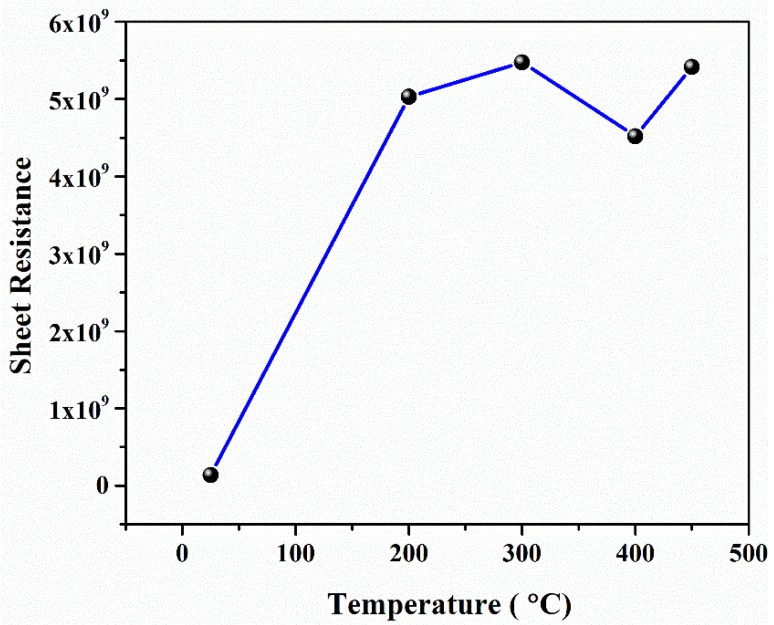
Sheet resistance of PbS thin film.

The sheet resistance can be determined by the following formula [39,40,41]:

(2) $$R_{s}=\frac{\;\rho }{t}$$

where “ρ” is the resistivity of the material and “*t*” is the thickness of thin film. The graphical results in Figure 8 show that sheet resistance exhibits exactly the opposite behavior to that of conductivity due to the reasons mentioned earlier.

## 4. Conclusions

In the present study, a thin film of PbS deposited via the e-beam evaporation method onto soda lime glass was crystalline in nature.

- The films were deposited with an intended thickness of 150 nm and were physically stable, pinhole-free, and typically had better adhesion property with good uniformity.
- A sufficient amount of impurity was instinctively added due to annealing the film in an open atmosphere. As a result, an insignificant amount of oxygen in annealed samples reacted with lead and became lead oxide, which was confirmed by XRD.
- Rutherford backscattering spectrometry confirmed the thickness of film (172 nm) was slightly greater than the intended thickness of 150 nm with lead and sulfur in the films (along with substrate concentration). The diffused oxygen due to annealing shows that the concentration of lead decreases with the increase in the amount of oxygen. In contrast, a comparatively slight difference was found in EDS results due to the limitations of SEM. Both results confirmed that the oxygen amount increases with an increase in annealing temperature.
- The transmittance of as-deposited film is lower, as the annealing temperature increases; similarly, the transmittance of the film drastically increases at a shorter wavelength. This is due to the reduced light scattering at a higher temperature. A band gap of PbS film is highly influenced by temperature. The *E*_g_ of PbS films was determined to be in the 2.12–2.75 eV range. Hence, band gap can be tuned in order with the respective application for IR applications.
- PbS is a p-type semiconducting material determined by Hall measurements. The film’s sheet resistance is greatly increased with an increase in annealing temperature; alternately, a larger reduction in career concentration and high mobility has been found. As-deposited PbS thin film shows good conductivity, which is suitable for optoelectronic applications.

In summary, it was found that composite PbS along with PbO thin films show unique properties that can be employed over a variety of applications such as optoelectronic circuits, optical circuits, and infra-red detectors. The obtained results of thin film over different temperature ranges showed different band gaps, which may be suitable for various applications, depending on the requirement range of the device. However, the industry needs smart and sensitive detectors for actuators; for this purpose, the work on the composite PbS along with PbO thin films was quite useful.

## Figures and Tables

**Table 1:** Elemental concentrations determined by EDS of as-deposited PbS and annealed thin film at 200 °C, 300 °C, 400 °C, and 450 °C, respectively.

Sample 1 **PbS at 25** °C	**Elements**	**Weight %**	**Atomic %**
	O	6.45	29.0
	Si	11.3	29.0
	Mg	0.56	1.54
	S	5.45	12.2
	Ca	1.14	2.16
	Pb	75.1	26.1
**Total**		**100.00**	**100.00**
Sample 2 **PbS at 200** °C	**Elements**	**Weight %**	**Atomic %**
	O	7.01	31.0
	Si	11.9	30.0
	Mg	0.41	1.05
	S	4.38	9.7
	Ca	1.5	2.65
	Pb	74.8	25.6
**Total**		**100.00**	**100.00**
Sample 3 **PbS at 300** °C	**Elements**	**Weight %**	**Atomic %**
	O	12.8	46.8
	Si	12.9	26.9
	Mg	0.5	1.21
	S	1.3	2.38
	Ca	1.90	2.71
	Pb	70.6	20.0
**Total**		**100**	**100**
Sample 4 **PbS at 400** °C	**Elements**	**Weight %**	**Atomic %**
	O	8.46	35.45
	Si	12.72	30.36
	Mg	0.44	1.21
	S	3.1	6.48
	Ca	1.59	2.66
	Pb	73.69	23.84
**Total**		**100.00**	**100.00**
Sample 5 **PbS at 450** °C	**Elements**	**Weight %**	**Atomic %**
	O	11.51	43.78
	Si	13.15	28.49
	Mg	0.47	1.19
	S	1.48	2.81
	Ca	1.78	2.70
	Pb	71.61	21.03
**Total**		**100.00**	**100.00**

## Data Availability

The data presented in this study are available on request from the corresponding author.
